# Small interfering RNA-induced inhibition of epithelial Cell transforming sequence 2 suppresses the proliferation, migration and invasion of osteosarcoma cells

**DOI:** 10.3892/etm.2021.10408

**Published:** 2021-07-09

**Authors:** Jie Xie, Pengfei Lei, Yihe Hu

Exp Ther Med 9:1881–1886, 2015; DOI: 10.3892/etm.2015.2306

Following the publication of this paper, it was drawn to the Editors' attention by a concerned reader that certain of the flow cytometric data shown in Fig. 3 and Transwell cell migration data shown in Fig. 4A and B were strikingly similar to data appearing in different form in other articles by different authors. Owing to the fact that the contentious data in the above article had already been published elsewhere, or were already under consideration for publication, prior to its submission to *Experimental and Therapeutic Medicine*, the Editor has decided that this paper should be retracted from the Journal. The authors were asked for an explanation to account for these concerns, but the Editorial Office did not receive any reply. The Editor apologizes to the readership for any inconvenience caused.

